# Cardiac fibrosis can be attenuated by blocking the activity of transglutaminase 2 using a selective small-molecule inhibitor

**DOI:** 10.1038/s41419-018-0573-2

**Published:** 2018-04-27

**Authors:** Zhuo Wang, Daniel J. Stuckey, Colin E. Murdoch, Patrizia Camelliti, Gregory Y. H. Lip, Martin Griffin

**Affiliations:** 1 School of Life and Health Sciences, Aston University, Aston Triangle, Birmingham, B4 7ET UK; 2 Centre for Advanced Biomedical Imaging, University College, London, WC1E 6DD UK; 3 Aston Medical Research Institute, Aston Medical School, Aston University, Aston Triangle, Birmingham, B4 7ET UK; 4 School of Biosciences and Medicine, Faculty of Health and Medical Sciences, University of Surrey, Guildford, GU2 7XH UK; 5 Institute of Cardiovascular Sciences, City Hospital, Birmingham, B18 7QH UK

## Abstract

Cardiac fibrosis is implicit in all forms of heart disease but there are no effective treatments. In this report, we investigate the role of the multi-functional enzyme Transglutaminase 2 (TG2) in cardiac fibrosis and assess its potential as a therapeutic target. Here we describe the use a highly selective TG2 small-molecule inhibitor to test the efficacy of TG2 inhibition as an anti-fibrotic therapy for heart failure employing two different in vivo models of cardiac fibrosis: Progressively induced interstitial cardiac fibrosis by pressure overload using angiotensin II infusion: Acutely induced focal cardiac fibrosis through myocardial infarction by ligation of the left anterior descending coronary artery (AMI model). In the AMI model, in vivo MRI showed that the TG2 inhibitor 1–155 significantly reduced infarct size by over 50% and reduced post-infarct remodelling at 20 days post insult. In both models, Sirius red staining for collagen deposition and levels of the TG2-mediated protein crosslink ε(γ-glutamyl)lysine were significantly reduced. No cardiac rupture or obvious signs of toxicity were observed. To provide a molecular mechanism for TG2 involvement in cardiac fibrosis, we show that both TGFβ1-induced transition of cardiofibroblasts into myofibroblast-like cells and TGFβ1-induced EndMT, together with matrix deposition, can be attenuated by the TG2 selective inhibitor 1–155, suggesting a new role for TG2 in regulating TGFβ1 signalling in addition to its role in latent TGFβ1 activation. In conclusion, TG2 has a role in cardiac fibrosis through activation of myofibroblasts and matrix deposition. TG2 inhibition using a selective small-molecule inhibitor can attenuate cardiac fibrosis.

## Introduction

Heart disease remains the leading cause of death worldwide and its prevalence is likely to increase further with changes in lifestyle and as the population ages. Therefore, there is an urgent need for new drugs that are effective in treating heart disease patients. Cardiac fibrosis is implicit in all forms of heart disease. Fibrosis is a scarring process characterised by myofibroblast accumulation and excessive deposition of extracellular matrix (ECM) proteins, in particular collagen I and III. This can lead to loss of organ architecture and compliance, induction of pathological signalling in cardiomyocytes and eventual heart failure. The fibrotic process is similar in many organs, including lung, liver and kidneys, making it an attractive target for therapeutic intervention. However, targeted therapy is complicated, as the fibrosis causing myofibroblasts can originate from multiple cell types including endothelial cells (ECs) (known as endothelial–mesenchymal transition, EndMT)^1^, pericytes^2^, epithelial cells (epithelial–mesenchymal transition, EMT)^3^ and fibroblasts^4^. During cardiac fibrosis, genetic lineage studies indicate that the majority of myofibroblasts result from resident cardiofibroblasts^5^. However, EndMT-derived myofibroblasts may also play an important role^1,6^, particularly in the loss of resident blood vessels, in the area of tissue damage via capillary rarefaction^7^.

The transforming growth factor β (TGFβ) family of growth factors are pivotal in driving the transition of fibroblasts, endothelial cells, pericytes and epithelial cells into active myofibroblast in response to fibrotic stimuli^8–10^. The most documented member associated with fibrosis development is TGFβ1. Mature TGFβ1 is part of a latent complex consisting of a TGFβ1 dimer non-covalently bound to its latency-associated peptide (LAP), which is associated to a large TGFβ-binding protein (LTBP). Once activated, TGFβ1 binds to its ubiquitously expressed cell surface TGFβ1 type I and type II receptors, leading to the activation of a downstream signalling cascade involving both canonical, e.g., phosphorylation of Smad proteins and non-canonical signalling. This leads to the transcriptional regulation of a range of genes involved in the transition of cells into myofibroblasts leading to increased matrix deposition and fibrosis.

Transglutaminase 2 (TG2) is a multi-functional Ca^2+^-dependent protein crosslinking enzyme, which is regulated by TGFβ1 and also involved in the activation of matrix-bound latent TGFβ^11^. Proof-of-concept studies using animal models indicate TG2 is involved in lung^12^ and kidney fibrosis^13^, where it has a role in matrix deposition and accumulation, and in latent TGFβ1 activation. However, transglutaminase inhibitors were only used in kidney fibrosis models and the inhibitors used in these studies were not selective for TG2^13^. We have now developed via the aid of in silico modelling highly potent TG2 selective inhibitors capable of reducing angiotensin II (AngII)-induced nephrosclerosis in mice^14^. We demonstrated a mechanism for the highly potent cell permeable compound 1–155 whereby the inhibitor reduced both TG2 activity and export of TG2 into the ECM by blocking its cell surface interaction with its binding partner syndecan-4^13^, which is required for TG2 secretion^15^.

Given our earlier preliminary data suggesting the importance of TG2 in kidney fibrosis^14^, we sought to clarify its role in the development of cardiac fibrosis using for the first time an inhibitor that is selective for TG2. Our data obtained from both cell models and two well-characterised in vivo models indicate a role for TG2 in the development of cardiac fibrosis. We demonstrate that the TG2 selective inhibitor 1–155 reduces fibrosis in vitro and in two clinically relevant mouse models of cardiac fibrosis, making TG2 an attractive drug target for anti-fibrotic therapies.

## Results

### The effects of TG2 inhibition in mouse models of cardiac fibrosis

#### Angiotensin II model of progressive interstitial cardiac fibrosis

**Fig. 1:**
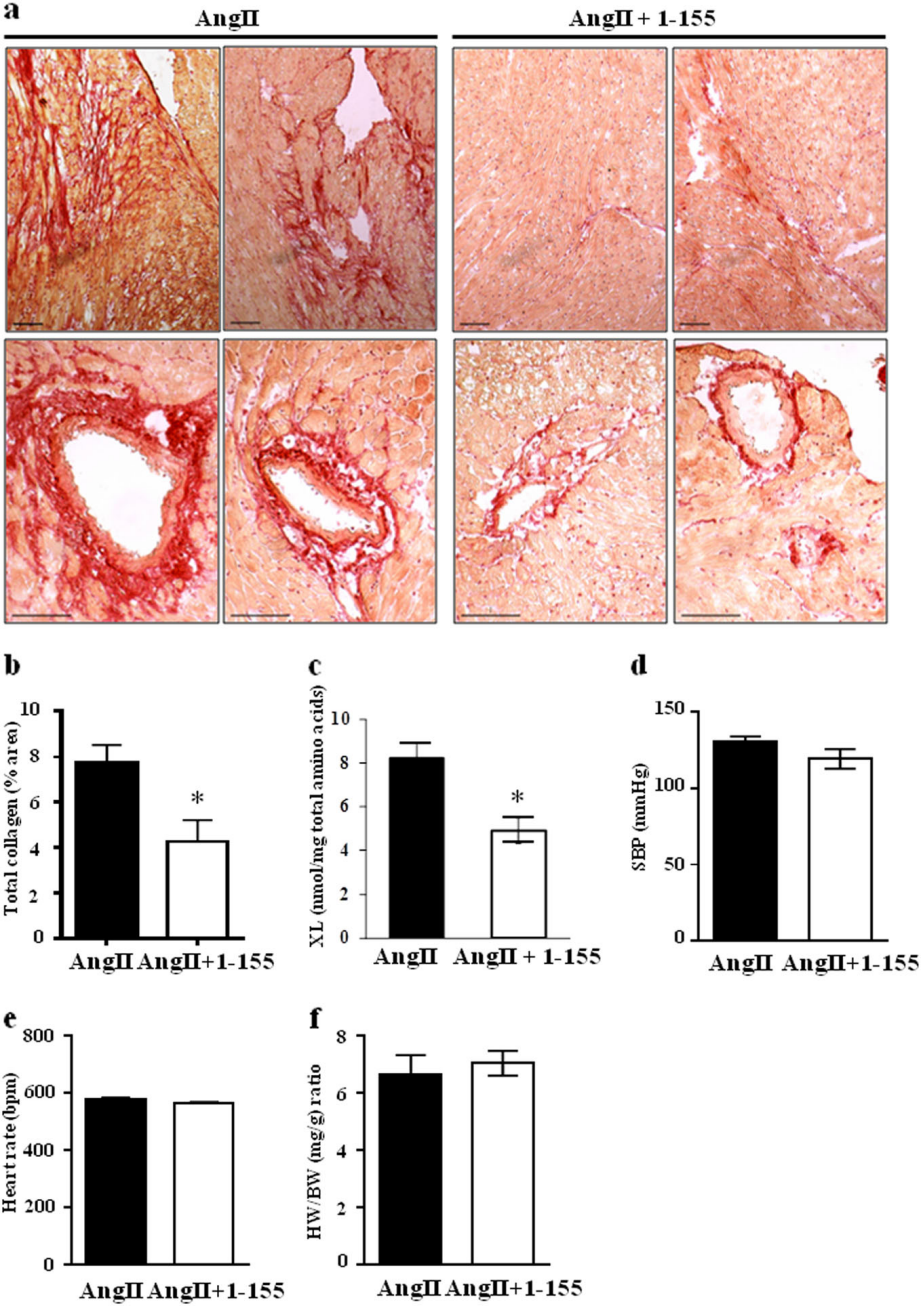
In vivo assessment of TG2 inhibition on AngII-induced cardiac fibrosis. AngII 1.1 mg/kg/day in 50% DMSO in PBS pH 7.4 was used to induce cardiac fibrosis for 2 weeks with or without TG2 inhibitor (25 mg/kg/day) via a subcutaneously implanted mini-pump (Alzet1002). **a** Representative images of heart sections from the inhibitor and non-inhibitor-treated animals showing collagen staining using Picro-Sirius red. Bar = 100 µm. **b** Averaged data of Picro-Sirius red/collagen staining in heart sections from control and TG2 inhibitor 1–155 treated animals. **c** TG2-mediated ε(γ-glutamyl)lysine crosslink (XL) formation in the AngII and AngII+ 1–155 treated animals. **d–f** Systolic blood pressure (SBP) (**d**), heart rate (**e**), and heart weight (HW, wet weight, mg) against body weight (BW, **g**). **f** were obtained after 2 weeks of treatment with 1–155. Data are means ± SE. *n* = 5/4. *p < 0.05

To demonstrate the importance of TG2 crosslinking activity in cardiac fibrosis and to validate TG2 as a potential therapeutic target, we first looked at a progressive diffuse model of cardiac fibrosis, where hypertension was induced by chronic infusion of angiotensin II (AngII), which provides an example of reactive fibrosis. AngII and TG2 inhibitor 1–155 were delivered over 14 days using a subcutaneously implanted osmotic pump. Histological staining using Picro-Sirius Red indicated mice receiving AngII plus vehicle control showed collagen deposition around the arterioles and in the heart interstitium (Fig. 1a). In contrast, animals receiving AngII plus TG2 inhibitor 1–155 showed significantly less (~50%) collagen deposition in all heart regions (Fig. 1a, b). Proof-of-target engagement by the TG2 inhibitor 1–155 was shown by a significant (~50%) reduction in the TG2-mediated crosslink ε-(γ-glutamyl)-lysine (Fig. 1c) when compared to the AngII control mice. TG2 inhibitor 1–155 was deemed to be directly targeting cardiac fibrosis since AngII plus TG2 inhibitor 1–155 had no effect on systolic blood pressure (Fig. 1d), heart rate (Fig. 1e) and heart weight body weight ratio (Fig. 1f) compared to AngII plus vehicle after 14 days of treatment.

#### Acute myocardial infarction model of focal cardiac fibrosis

**Fig. 2:**
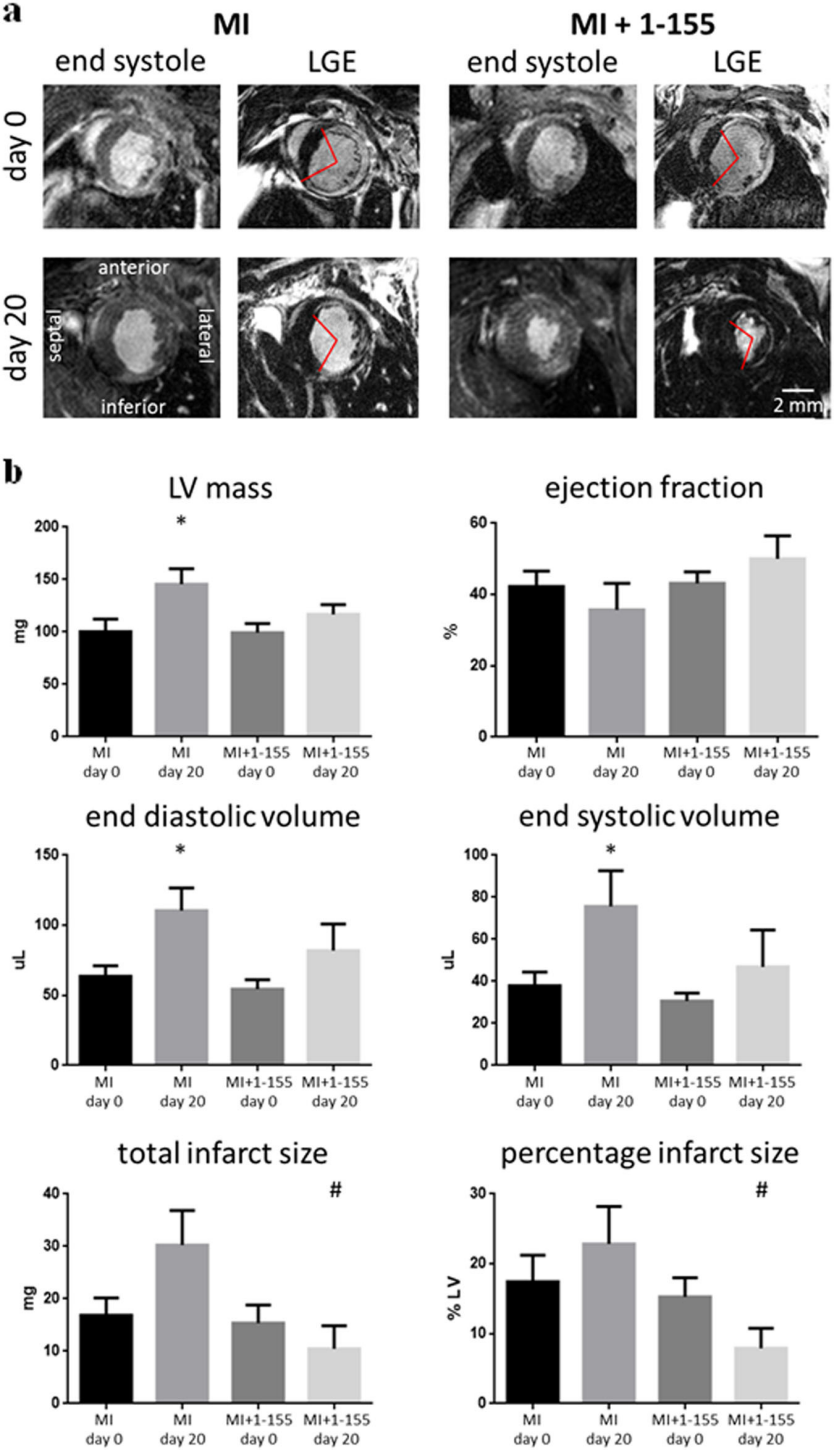
In vivo assessment of cardiac structure, function and viability in a mouse AMI model following TG2 inhibition. MRI was performed 6 h after myocardial infarction, prior to mini-pump implantation, and again at 20 days. **a** Representative end systolic frames of cine-MRI acquisitions along with matching late gadolinium-enhanced MRI (LGE) acquisitions used for assessment of infarct size. Red lines define the edges of the hyper-enhanced infarct region. **b** Left ventricular masses, end diastolic volumes and end systolic volumes increased from day 0 to day 20 in the control group, but there was no significant increase in these parameters in the treatment group. Total infarct size and infarct size as a percentage of left ventricular mass was significantly lower in the treatment group compared with controls at 20 days. *P < 0.05 compared with day 0; ^#^*P* < 0.05 compared with control. Bar = 2 mm

**Fig. 3:**
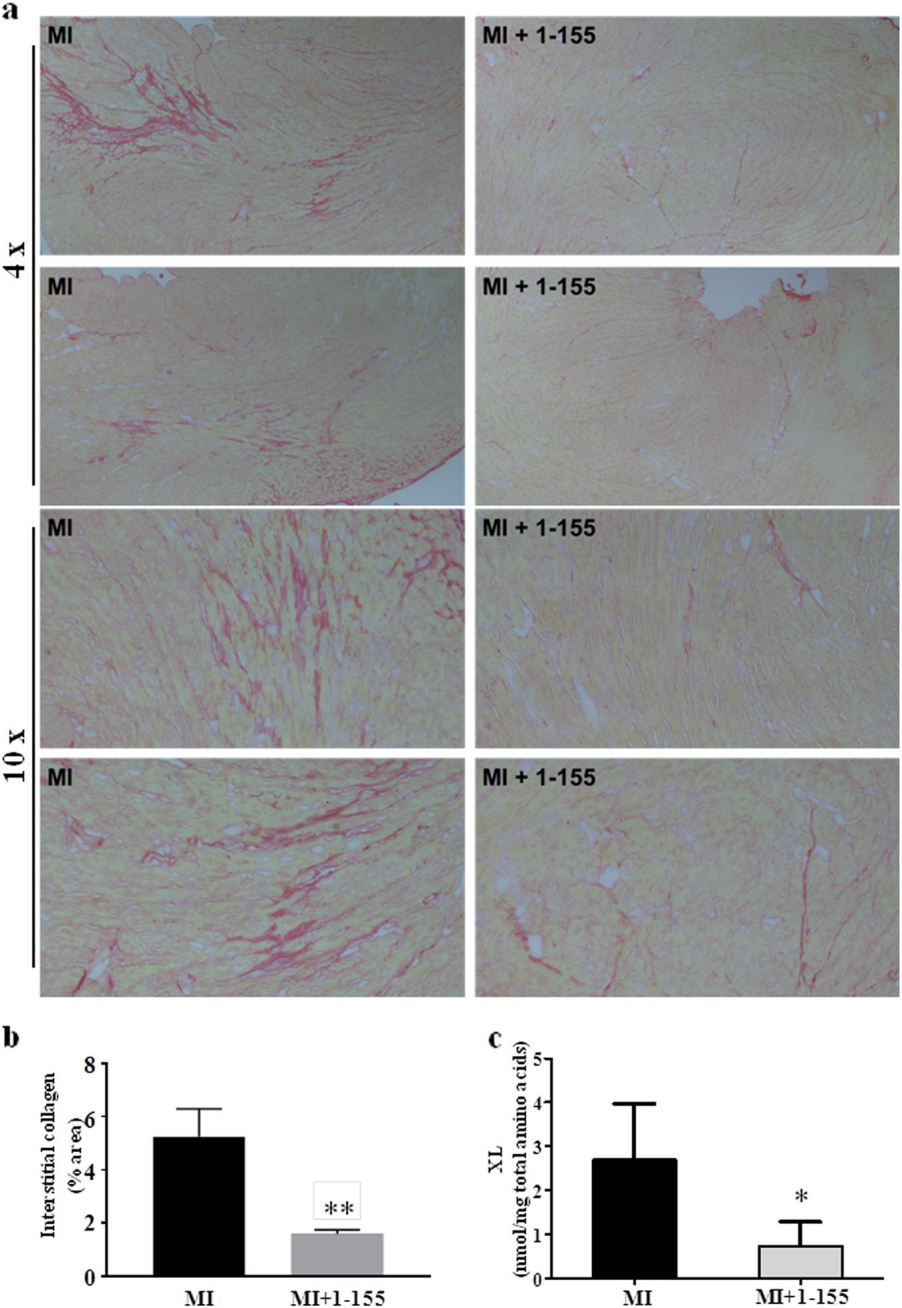
Assessment of collagen and TG2-mediated ε-(γ-glutamyl)-lysine in the AMI mouse model following TG2 inhibition. **a** Representative images of Picro-Sirius red/collagen-stained heart sections from the AMI mouse model following treatment with or without TG2 inhibitor 1–155. **b** Averaged data of Picro-Sirius red/collagen staining in the remote myocardium showing interstitial collagen deposition is significantly reduced by 1–155. **c** 1–155 reduces the TG2-mediated ε-(γ-glutamyl)-lysine crosslink (XL) in the 1–155 treated mice. Data are means ± SE. *n* = 5/7. *p < 0.05. **p < 0.005

The effects of TG2 inhibition on cardiac fibrosis were further tested in a mouse model of acute myocardial infarction, where replacement fibrosis occurs in the infarct region and interstitial fibrosis in the remote myocardium. Baseline cardiac function and infarct size was assessed after 6 h using in vivo MRI. These data were used to match cardiac impairment in the treated and control groups at baseline, prior to therapy (Fig. 2a). TG2 inhibitor 1–155 or vehicle control was delivered over 14 days, using subcutaneously implanted osmotic pumps inserted at 24 h after infarction. MRI was repeated at 20 days and followed by histology and analysis of the TG2-mediated crosslink ε-(γ-glutamyl)-lysine in heart tissue. Baseline cardiac structure, function and infarct size were similar between groups (Fig. 2a, b). During the follow-up period, one animal died in each group and no evidence of cardiac rupture was identified upon autopsy. From day 0 to day 20 the left ventricular masses, end diastolic volumes and end systolic volumes increased in the control group as is expected in this model, but there was no significant increase in these parameters in the group treated with TG2 inhibitor 1–155 (Fig. 2b). Ejection fraction was higher in the group treated with TG2 inhibitor 1–155 compared with controls at 20 days, but this difference was not significant (Fig. 2b). Total infarct size and infarct size as a percentage of left ventricular mass was significantly lower in the group treated with TG2 inhibitor 1–155 compared with vehicle controls at 20 days. Histological staining using Picro-Sirius Red identified significantly less interstitial collagen deposition in the remote myocardium of the mice treated with 1–155 (Fig. 3a), confirming the compound’s ability to reduce collagen deposition in vivo (Fig. 3b). Similarly, measurement of TG2-mediated crosslink ε-(γ-glutamyl)-lysine in the remote myocardial tissue of the mice treated with 1–155 indicated a comparable and significant reduction confirming target engagement of the TG2 inhibitor (Fig. 3c).

### TG2 in the TGFβ1-induced transition of cardiofibroblasts into myofibroblasts

**Fig. 4:**
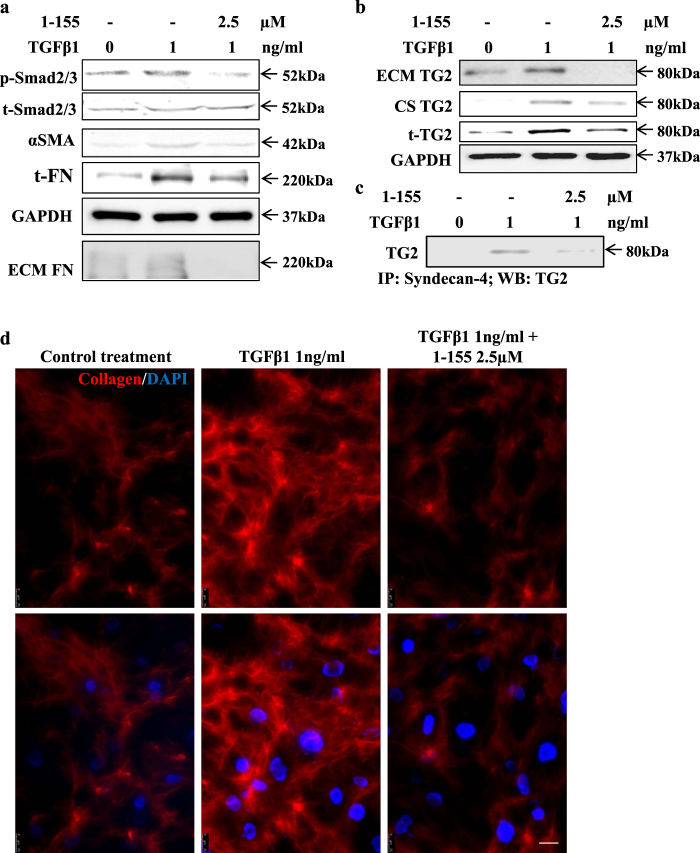
The effect of TG2 inhibition on TGFβ1-induced cardiofibroblast to myofibroblast transition and collagen deposition in human cardiofibroblasts. **a** Representative western blots (*n* = 3) showing p-Smad2/3 activation, αSMA and FN and FN deposition and the inhibition of these parameters using TG2 inhibitor 1–155 (2.5 µM) in 72 h TGFβ1-treated cardiofibroblasts at the concentrations shown. Matrix FN was measured according to the procedures described in the 'Materials and methods'. **b** Representative western blot (*n* = 3) showing the effect of 72 h TGFβ1 treatment in cardiofibroblasts showing increased TG2 expression and increased TG2 at the cell surface and ECM and inhibition of this by TG2 inhibitor 1–155. **c** Representative western blot (*n* = 3) of TG2 in the syndecan-4 immunocomplex from co-IP carried out as described in 'Materials and methods'. **d** Immunofluorescence detection of collagen I in cardiofibroblasts treated with 1 ng/ml TGFβ1 over 5 days with and without the TG2 inhibitor at 2.5 µM undertaken as described in the 'Materials and methods'. Bar = 25 µm

Cardiofibroblasts are the major source of myofibroblasts during the development of cardiac fibrosis; therefore, in order to provide a molecular mechanism for the involvement of TG2 in cardiac fibrosis we first looked at the importance of TG2 in TGFβ1-treated human cardiofibroblasts. Treatment of human cardiofibroblasts with TGFβ1 led to the activation of the Smad2/3 signalling, resulting in a significant increase in fibronectin expression and matrix deposition, and a significant increase in expression of myofibroblast marker SMAα, indicating their transition into activated fibroblasts (Fig. 4a and Supplementary Figure S1). When cells were incubated with the TG2 selective inhibitor 1–155 (which gave rise to no obvious signs of cell toxicity, Supplementary Figure S2), there was a significant reduction in these myofibroblast markers (Fig. 4a and Supplementary Figure S1). We have previously shown that in NIH3T3 fibroblasts, TG2 externalisation is dependent on its direct interaction with the cell surface heparan sulphate proteoglycan syndecan-4, a process which can be blocked by the cell permeable selective TG2 inhibitor 1–155^13^. Here, we next studied TG2 secretion in cardiofibroblasts and showed that TGFβ1 treatment led to a significant increase in TG2 expression and externalisation to the cell surface and ECM, which could be significantly reduced by 1–155 (Fig. 4b and Supplementary Figure S3). The TG2 selective inhibitor 1–155 was able to block TG2 expression and externalisation after TGFβ1 treatment by reducing TG2 interaction with syndecan-4 (Fig. 4c and Supplementary Figure S4). Importantly, inhibition of TG2 activity and its deposition into the matrix by 1–155 led to a significant reduction of the amount of collagen I deposited by cardiofibroblasts following treatment with TGFβ1 (Fig. 4d and Supplementary Figure S5).

### The induction of EndMT by TGFβ1 and its reversal by TG2 inhibitor 1–155

**Fig. 5:**
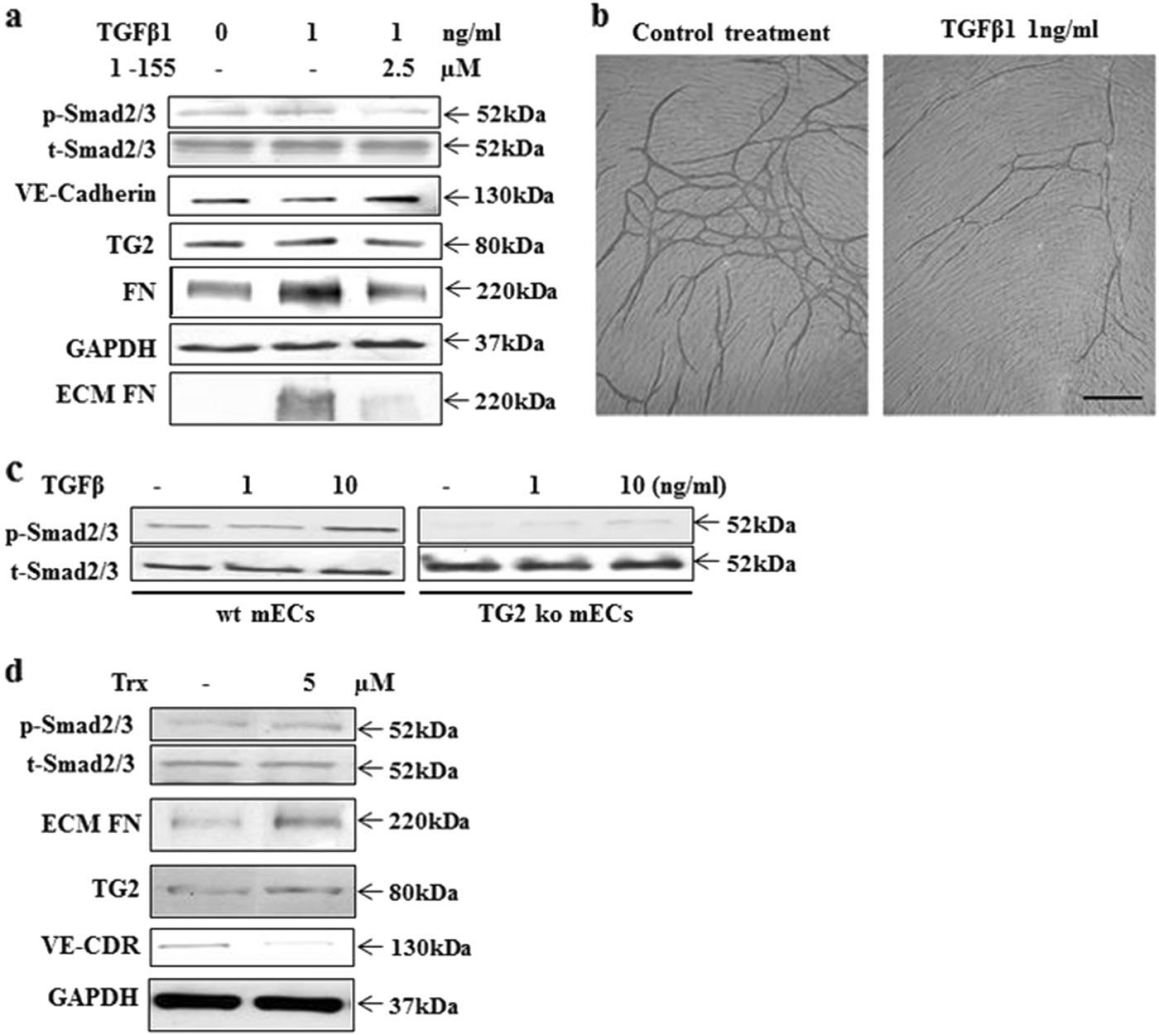
The importance of TG2 on TGFβ1-mediated EndMT. **a** Representative western blot (*n* = 3) showing p-Smad2/3 activation, VE-cadherin and FN expression, and FN deposition into the matrix in HUVECs treated with TGFβ1 in the presence or absence of t TG2 inhibitor 1–155. **b** The V2a AngioKit co-culture model was used as described in the 'Materials and methods' to study the effects of TGFβ1 on endothelial tubule formation. Representative images show the inhibitory effects of TGFβ1 on tubule formation at the concentration shown over 12 days. **c** Representative western blot (*n* = 3) showing Smad2/3 activation in mouse TG2^+/+^ and TG2^−/−^ microvascular ECs following TGFβ1 treatment for 72 h at the concentrations shown. **d** Representative western blot (*n* = 3) showing the effects of thioredoxin treatment on HUVECs showing Smad2/3 activation, expression of FN, VE-Cadherin and TG2

We next looked at the role of TG2 in myofibroblast formation through TGFβ1-induced EndMT. The well-characterised human umbilical vein endothelial cells (HUVECs) and primary TG2 knockout (TG2^−/−^) and wild-type (TG2^+/+^) mouse microvascular ECs isolated from the lungs were used in our experiments. Addition of TGFβ1 at 1 ng/ml to HUVECs led to an increase in Smad2/3 signalling with significant increases in fibronectin (FN) expression and matrix deposition, a small increase in TG2 expression and a significant reduction in the endothelial marker VE-cadherin, indicating the induction of EndMT. Addition of TG2 inhibitor 1–155, which is not toxic to HUVECs at the concentrations used^13^, reversed the effects of exogenous TGFβ1 as indicated by a significant reduction in p-Smad signalling, FN expression and deposition and the return of the endothelial cell marker VE-cadherin (Fig. 5a and Supplementary Figure S6). The addition of TGFβ1 to HUVECs also led to a parallel loss of endothelial tubule formation (Fig. 5b and Supplementary Table S2). Confirmation of the requirement for TG2 in TGFβ1 signalling needed for the induction of EndMT was indicated by use of mouse lung TG2^−/−^ ECs. Stimulation of the TG2^+/+^ ECs with mouse TGFβ1 resulted in induction of Smad2/3 signalling, which was significantly reduced in the TG2^−/−^ ECs even at high TGFβ1 concentration of 10 ng/ml (Fig. 5c and Supplementary Figure S7).

Recent evidence has indicated the importance of thioredoxin in the activation of matrix-bound latent-oxidised TG2 during tissue injury and wound healing^16^. We show that exogenous addition of thioredoxin to HUVEC cells leads to the activation of TGFβ1-mediated Smad2/3 signalling with a significant increase in FN deposition, TG2 and decreased expression of VE-cadherin indicative of EndMT (Fig. 5d and Supplementary Figure S8).

## Discussion

Despite continuous progress towards risk factor reduction, heart disease remains the leading cause of death in the industrialised world. Present treatments do little to prevent the underlying development of fibrosis and mainly aim at preventing adverse long-term sequelae^17^. Moreover, there are few drugs presently undergoing clinical trials for attenuation of cardiac fibrosis.

The multi-functional protein crosslinking enzyme TG2 has been reported to have a role in lung and kidney fibrosis, but its function in cardiac fibrosis is still unexplored. Compared to lung and kidney, the cell composition of heart is unique being composed mainly of cardiomyocytes with respect to volume, fibroblasts, endothelial cells and a smaller number of pericytes^18^. Cardiac fibrosis can manifest itself in two different forms, reactive fibrosis and replacement fibrosis^19^. For example, hypertension and pressure overload in the heart from clinical aortic stenosis or preclinical administration of angiotensin II gives rise to reactive fibrosis, which progresses without significant loss of cardiomyocytes^20^. In contrast in acute myocardial infarction (AMI), ischaemia leads to initial cell death, followed by an inflammatory reaction with replacement fibrosis in the infarct region and progressive reactive fibrosis in the remote myocardium^21^. In both cases, the major sources of myofibroblasts are the resident cardiofibroblasts with ECs also making a contribution^4^.

In this report, we have explored the promise of TG2 as a potential therapeutic target in the treatment of cardiac fibrosis with a highly selective TG2 inhibitor^14^, using two different preclinical models representative of the reactive and replacement fibrosis that is most frequently found in heart disease. In addition, in order to understand why TG2 might be a good therapeutic target in cardiac fibrosis, we explored the role of TG2 in myofibroblast development using two cell models consisting of cardiofibroblasts and ECs^4^.

In the first preclinical model we used a progressive reactive model of cardiac fibrosis, where hypertension was induced by chronic infusion of AngII with one group of mice receiving the TG2 selective inhibitor 1–155 and the other group vehicle alone. Following a 14-day delivery of AngII by an osmotic pump implanted subcutaneously, collagen deposition was found around the major blood vessels (perivascular) and in the interstitium (interstitial) of the mouse hearts, which could be significantly reduced by around 50% in the mice receiving the TG2 inhibitor 1–155, indicating that the reactive fibrosis induced in this model has been attenuated.

Importantly, this observation of a reduction in deposited collagen which when crosslinked by TG2 prevents turnover of the fibrotic collagen matrix mirrors that found in a recent publication using the transverse aorta constriction model (TAC) in TG2 ko mice^22^. In this TG2 ko model in the wt control mice increased TG2 expression was found in cardiomyocytes, interstitial cells and in the extracellular matrix, but increases in TG2 activity as defined by an increase in ε(γ-glutamyl) lysine crosslink was not measured. In a further very recently published TAC model, using wt mice on a C57BL/6J background mice were treated with the transglutaminase inhibitor ERW1041E ((S)-Quinolin-3-ylmethyl 2-((((S)-3-Bromo-4, 5-dihydroisoxazol-5-yl)methyl)carbamoyl)pyrrolidine-1-carboxylate) administered twice daily by intraperitoneal injection over 28 days^23^. In each of these animal models, whether it be TAC or AngII treated the insult results in progressive diffuse cardiac fibrosis which is reduced by TG2 ko or by inhibition of TG2. However, they are difficult to compare directly since in one, TG2 protein and activity is totally absent while in the preclinical models it is very likely that some TG2 activity is still present as is the TG2 protein, which may be important given the multi-functional roles of TG2 as demonstrated in the recent TAC model reported by Shinde et al.^23^

Notably in our AngII model, TG2 inhibitor 1–155 had no effect on systolic hypertension or heart rate when compared to the mice receiving AngII alone, indicating that TG2 inhibitor 1–155 was acting directly to prevent cardiac fibrosis in vivo, and not via a secondary change in blood pressure. In the AngII model, cardiac hypertrophy was also preserved, an adaptive component of LVH which is known to be protective in hypertensive settings^24^. Interestingly, in the TAC model, administration of the TG2 inhibitor ERW1041E had a protective effect on systolic function in male mice but not female mice although preserved diastolic function was noted in both genders. The authors also observed an increase in ejection fraction in sham animals receiving vehicle or ERW1041E, which they concluded was due to the multiple injection regime received by these animals^23^.

Comparison of the two preclinical models is also made difficult by the method of inhibitor administration, the selectivity of the inhibitor used and the methods used to show a reduction in TG2 activity. ERW1041E, unlike 1–155, is not highly selective for TG2 since its inhibition of TG1 is comparable to that of TG2^25^. Using intraperitoneal injection of this inhibitor twice a day can also cause problems as noted by the authors, unlike administration via mini-pump infusion, where PK values of the inhibitor also allow approximate calculations to be made on the steady-state concentration of the inhibitor present in the animal. In addition, in the TAC model, the authors used biotinylated pentylamine to assess target engagement by measuring in situ TG2 activity but this amine substrate is not specific for TG2 and measures all in situ transglutaminase activity. This is particularly important if the transglutaminase inhibitor used is not highly selective for TG2.

In our second study on TG2 involvement in cardiac fibrosis, the mouse model of myocardial infarction was used to induce replacement fibrosis in the infarct region and interstitial fibrosis in the remote myocardium. In vivo MRI was used to match baseline infarct sizes between groups acutely after infarction and prior to treatment. At 24 h after infarction, one group of mice received mini-pump infusion of the TG2 selective inhibitor 1–155 and the other group vehicle alone. At 20 days, mice treated with 1–155 had smaller infarct sizes, less ventricular remodelling and reduced interstitial fibrosis within the remote myocardium compared with controls. Importantly, no increased mortality was observed in the 1–155 treated mice, suggesting that treatment with our TG2 inhibitor allows for sufficient collagen deposition to prevent acute cardiac rupture.

In both the AngII and AMI models, treatment with the selective TG2 inhibitor 1–155 led to a significant reduction in cardiac fibrosis, indicating that the TGFβ1-induced pathways leading to myofibroblast formation and collagen deposition have been significantly attenuated. In the AMI model, the reduction in fibrosis was paralleled by a significant reduction in infarct size and remodelling.

Based on a measured in vivo, PK mouse Clp of >5.4 L/h/kg for 1–155 and a wide distribution of this cell permeable inhibitor in the mouse, the steady-state concentration of inhibitor in both models was calculated as ~0.35 µM. Given the EC50 for inhibition of fibronectin deposition in mouse cells is ~0.45 µM for 1–155^13^, our data demonstrate the efficacy of 1–155 in blocking fibrosis by TG2 inhibition. Importantly, in both models, target engagement by 1–155 leading to inhibition of TG2 was demonstrated by reduction in its crosslink ε-(γ-glutamyl)-lysine that paralleled the reduction in collagen deposition.

To provide a mechanism as to how inhibition of TG2 leads to a reduction of fibrosis in the two preclinical models, we first asked the question whether the increased levels of TGFβ1 found in the fibrotic heart which mediate differentiation of resident cardiofibroblasts into myofibroblasts can be inhibited by our selective TG2 inhibitor. Involvement of TG2 in TGFβ1 signalling is a new role for TG2 in the fibrotic mechanism since previous reports for the functional relationship between TG2 and TGFβ1 have focused on the activation of the matrix-bound latent form of TGFβ1^11^. Our data clearly indicate that inhibition of TG2 by its selective inhibitor 1–155 blocks exogenously added TGFβ1-induced Smad2/3 signalling leading to reversal of cardiofibroblast to myofibroblast transition. Hence, TG2 appears essential for TGFβ1 signalling in myofibroblast formation from cardiofibroblasts. This is in addition to its role in activating latent matrix-bound TGFβ1, both of which are key to the pathological role of TGFβ1 in fibrosis. We also demonstrate that incubation of cardiofibroblasts with TGFβ1 leads to an increased TG2 found both at the cell surface and in the ECM, which is essential for its role in matrix crosslinking and which could be inhibited by the TG2 inhibitor 1–155. The cell surface pool of TG2 was associated with the heparan sulphate proteoglycan syndecans-4, which is required in the translocation of TG2 into the ECM^15^. When TGFβ1-treated cardiofibroblasts were treated with the TG2 inhibitor 1–155, the association of TG2 with syndecan-4 was blocked due to the ability of 1–155 to react with TG2 and fix the enzyme in its open conformation preventing binding to syndecan-4 limiting translocation into the matrix, agreeing with our previous report using this inhibitor in NIH3T3 cells transduced with TG2^14^.

We cannot rule out that the reduced infarct size in the AMI model may also be due to increased survival of the cardiomyocytes following TG2 inhibition, although this seems unlikely given earlier reports indicating that in TG2^−/−^ mice, loss of TG2 leads to increased cardiomyocyte cell death. However, as stated earlier it is difficult to compare a TG2 knockout model with a preclinical model, where TG2 protein is still present and not all TG2 activity is likely to be inhibited^22^.

But in keeping with a role for TG2 in cardiac fibrosis earlier reports do indicate that targeted overexpression of TG2 in cardiomyocytes in mice leads to interstitial cardiac fibrosis^26^. In all the cardiac parameters, we measured in animals treated with TG2 inhibitor there is no suggestion from measurement of LV mass, ejection fraction, end systolic and end diastolic volume and infarct size that inhibition of TG2 causes increased apoptosis. In fact, our data suggest that TG2 inhibition leads to preservation of cardiomyocyte integrity.

EndMT induced by increases in TGFβ during the onset of fibrosis is reported to be a further important event in the fibrotic process^1^ shown in both kidney^27^ and cardiac fibrosis^28^. This process can lead to loss of existing endothelial cells, capillary rarefaction and inhibition of angiogenesis by the activated ECs in the fibrotic area as we have recently shown^29^. This cellular process can be demonstrated in vitro by addition of TGFβ1 to endothelial cells during tubule formation (Fig. 5b). Without new blood vessel growth, remodelling of the fibrotic area would as a consequence be difficult and prolonged. In addition to its role in EndMT, TG2 has been recognised to play a role in TGFβ1-induced EMT in cystic fibrosis^30^ and during cancer progression^31^, while our recent paper confirms its role in TGFβ1-induced EndMT^32^. Confirmation of a role for TG2 in canonical TGFβ1 signalling was shown by the significant reduction of TGFβ1-induced p-Smad signalling in mouse TG2^−/−^ endothelial cells. These observations explain our previous findings, where TG2 injection into tumour tissues blocked angiogenesis in a mouse colon carcinoma^3^.

Interestingly, addition of exogenous thioredoxin, a known activator of oxidised extracellular TG2, also led to EndMT in HUVEC cells. Thioredoxin is normally associated with a protective role during chronic inflammatory conditions^33^. It is therefore not unreasonable to suggest that activation of matrix-bound oxidised TG2 leading to matrix crosslinking is required in the initial wound healing response forming part of the protective role of thioredoxin prior to onset of progressive fibrosis in the later stages.

**Fig. 6:**
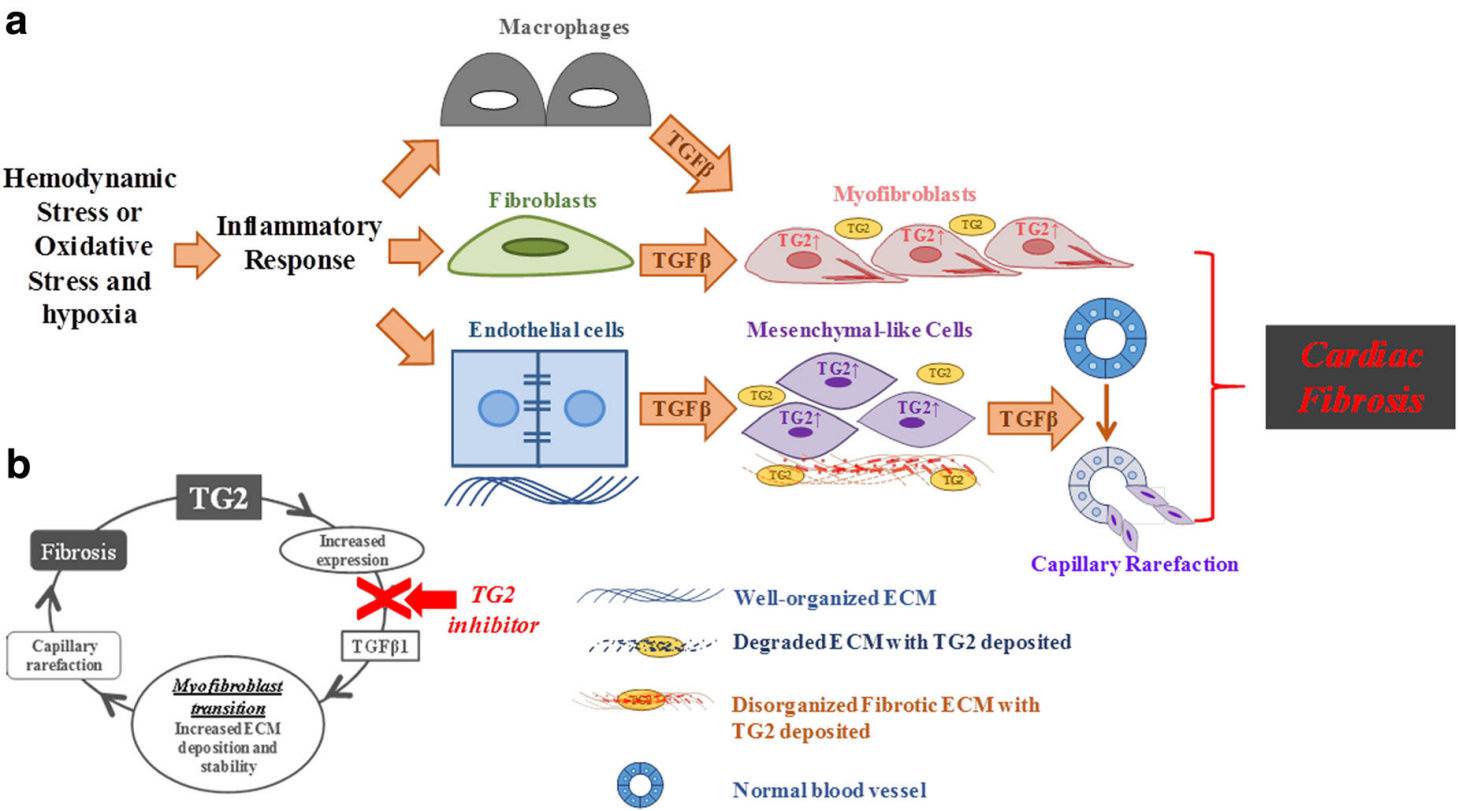
Schematic showing the role of TG2 in cardiac fibrosis and the TG2/TGFβ1 positive feedback mechanism indicating its importance in the progression of cardiac fibrosis. **a** How hemodynamic or oxidative stress and hypoxia can lead to an inflammatory response causing increased amounts of extracellular TGFβ1 leading to increased amounts of TG2 and increased numbers of myofibroblast leading to the deposition of a highly TG2 crosslinked fibrotic matrix. **b** The functional relationship between TG2 in the progression of fibrosis. The arrow shows how inactivation of TG2 can block this vicious cycle in fibrosis development

Once TG2 is active in the ECM either through thioredoxin reduction or via increased secretion, its crosslinking activity will lead to the remodelling of the ECM proteins, such as collagens. In addition, the involvement of TG2 in latent TGFβ1 activation, its requirement for TGFβ1 signalling and the ability of TGFβ1 to induce TG2-expressing myofibroblasts lead to a vicious positive feedback cycle, resulting in increased TGFβ1 increased TG2 and progressive fibrosis (Fig. 6). Our preclinical studies in two different models of cardiac fibrosis clearly indicate that this vicious positive feedback cycle can be blocked by selective inhibition of TG2 leading to a reduction in collagen deposition and fibrosis.

Our work therefore confirms the role of TG2 in cardiac fibrosis as demonstrated by its importance in the TGFβ1 induction of cardiac myofibroblasts from resident cardiofibroblasts and the induction of EndMT ultimately leading to increased matrix deposition and capillary rarefaction. We also show TG2’s ability to regulate TGFβ1 signalling in both endothelial cells and in cardiac fibroblasts, an observation which we recently demonstrated in cystic fibrosis bronchial epithelial cells^30^.

In conclusion, we demonstrate not only the importance of TG2 in cardiac fibrosis and the profound effect of its inhibitor 1–155 on attenuating the progression of cardiac fibrosis in two different models, but also mechanisms of the TG2-mediated fibrotic process, i.e., driving EndMT in endothelial cells and cardiofibroblast transition to myofibroblasts via TGFβ1 signalling. Emerging evidence suggests the potential of TG2 as a therapeutic target for various diseases and early clinical trials are taking place, e.g., diabetic nephropathy to test the effects of TG2 inhibition as a therapeutic strategy for this disease. Therefore, the significance of our work indicating the importance of TG2 as a novel and translational therapeutic target for the prevention of heart failure has a great potential in both scientific and clinical fields.

## Materials and methods

A detailed methodology is presented in the Supplementary Information-Methods.

### Reagents and antibodies

The general reagents were purchased from Sigma-Aldrich (UK), unless stated below. The peptidomimetic irreversible TG2 selective inhibitor 1–155 was synthesised at Aston University^14^. Antibodies used in this study are listed in Supplementary Information Table 1.

### In vitro studies

HUVECs were from Lonza (Germany). Human cardiofibroblasts were from PromoCell (Germany). TG2^−/−^ and control TG2^+/+^ ECs were isolated from 4–6 weeks old B6 TG2 ko or wt mice^32^. Details of well-established methods by our group used for in vitro studies, including the assay of cell viability, the V2a Angio kit angiogenesis co-culture system biotinylation of cell surface proteins, co-immunoprecipitation SDS-PAGE and western blotting, immunofluorescence staining are detailed in the Supplementary Information -Methods.

### In vivo studies

Two animal models of cardiac fibrosis were used in our study. A progressive diffuse model of cardiac fibrosis where hypertension was induced by chronic infusion of AngII leading to left ventricular hypertrophy and diffuse interstitial cardiac fibrosis and an AMI model of focal cardiac fibrosis induced after permanent ligation of the proximal left coronary artery. In the AMI model, in vivo imaging was used to provide serial quantification of cardiac function prior to and after myocardial infarction and therapy. Detailed experimental procedures and statistical analysis undertaken are detailed in the Supplementary Information-Methods.

For analysis of the ε(γ-glutamyl)lysine crosslink, tissue samples from both models were digested using a cocktail of proteolytic enzymes and the crosslink then analysed using cation exchange amino acid analysis using a lithium buffer system as previously described^14^.

### Statistical analysis

Unless stated otherwise, all values are presented as the mean ± SD for at least three independent replicate experiments (*n* ≥ 3). Data analyses were performed using either the Turkey and Dunnet test or the Student’s *t* test. A *p* value of less than 0.05 was considered to indicate statistical significance when is indicated in the text.

For animal work, statistical analysis of results was undertaken using the one-way analysis of variance (ANOVA) using a post test depending on the requirement. Data are expressed as the mean ± SE. A *p* value of less than 0.05 was considered to indicate statistical significance.

## Electronic supplementary material

Supplementary Files-Methods

Supplementary Files-Supplementary Figure 1

Supplementary Files-Supplementary Figure 2

Supplementary Files-Supplementary Figure 3

Supplementary Files-Supplementary Figure 4

Supplementary Files-Supplementary Figure 5

Supplementary Files-Supplementary Figure 6

Supplementary Files-Supplementary Figure 7

Supplementary Files-Supplementary Figure 8

Supplementary Files-Supplementary Table 1

Supplementary Files-Supplementary Table 2
