# Supplementary material for: Cardiac fibrosis can be attenuated by blocking the activity of transglutaminase 2 using a selective small-molecule inhibitor
Source: Cell Death Dis. 2018 Apr 27;9(6):613. doi: 10.1038/s41419-018-0573-2 (PMC5966415; doi:10.1038/s41419-018-0573-2)
Supplement: Supplementary file 1 — Supplementary Files-Methods [file 41419_2018_573_MOESM1_ESM.pdf]

Supplementary methods

Reagents and antibodies

The general reagents were purchased from Sigma-Aldrich (UK), unless stated below. The peptidomimetic irreversible TG2 inhibitor 1-155 was synthesized at Aston University [1]. Inhibitor 1-155 [Compound 3h as published [1]] is a highly selective cell permeable inhibitor of TG2. It has an IC50 of 6nM and a Kinact/Ki of 297,692 $min^{-1}M^{-1}$. Other pharmacological properties of 1-155 are documented previously [1]. The antibodies used in this work are listed in Supplementary Material **Table S2**.

***In vivo* studies-Experimental design**

Animal studies were undertaken with full ethical approval and all procedures carried out under license according to regulations laid down by Her Majesty's Government, United Kingdom (Animals Scientific Procedures Act, 1986) and undertaken in accordance with the 3Rs and ARRIVE guidelines.

1. Angiotensin II model of diffuse fibrosis

In vivo studies to assess the importance of TG2 in cardiac fibrosis were first performed in a model of hypertension induced by chronic infusion of Angiotensin II (AngII). This mouse model presents with left ventricular hypertrophy [2] and diffuse interstitial cardiac fibrosis. Further, it has been previously shown that AngII induced cardiac fibrosis can be linked to endothelial mesenchymal transition [3].

AngII and 1-155 was delivered as previously described [4, 5]. Fourteen week old male C57/BL/6 mice were implanted with osmotic pumps (Alzet1002) containing either AngII alone (1.1mg/kg/day in 50% DMSO in PBS, pH 7.4, n = 5) or AngII plus 25 mg/kg/day TG2 selective inhibitor 1-155 (1.1mg/kg/day AngII in 50% DMSO in PBS, pH 7.4, n = 5) 14 days. The steady state calculated concentration of 1-155 within the mice was approx. 0.35µM. This assumes a wide distribution of the inhibitor and is based on an *in vivo* PK Mouse Clp

>5.4L/h/kg. The cell EC50 for inhibition of fibronectin deposition in mouse cells was calculated as 0.45 µM with a comparable EC50 calculated for human cells [4].

Following 14 days, hearts were frozen in O.C.T (CellPath) at an optimal temperature (-150°C) using isopentane and 6µm sections were prepared. Picrosirius red staining of the left ventricle was performed to measure collagen staining as previously reported [2]. The percentage collagen content was quantified in a minimum of 10 images per mouse obtained from 7 sections.Sections were taken at the level of the valve using both control and inhibitor treated hearts. ImageProPlus (v5.0) was used to quantify the collagen staining. Other measurements taken from the treated mice included blood pressure (assessed by arterial pressure catheter under isoflurane anaesthesia), cardiomyocyte hypertrophy (assessed by left ventricle wet weight/ body mass ratio and wheat germ agglutinin staining of cardiomyocyte area). All secondary data points were determined at the start of the experiment. All experiments were conducted in a blind manner with no outliers or data excluded.

2. Acute Myocardial Infarction Model of focal cardiac fibrosis.

In the second model *in vivo* imaging was used to provide serial quantification of cardiac function prior to and after myocardial infarction and therapy, greatly enhancing the power of the results and reducing required sample sizes.

For coronary artery ligation C57BL/6 male mice (12 weeks old) were anaesthetised with 2% isofluorane and given buprenorphine (0.05 mg.kg-1) for analgesia. The trachea was intubated for mechanical ventilation (120 strokes.min-1, 200 µl stroke volume, Harvard Apparatus). The thorax was opened at the left 4th intercostal space and the proximal left coronary artery was permanently ligated using 8-0 Ethilon suture (Ethicon) [6].

In vivo MRI was performed at 6 hours and 20 days after coronary occlusion [7]. The baseline scans were used for quantification of infarct size prior to administration of therapy. This allowed infarct size to be matched between the treated and control groups at baseline and

permitted paired analysis of data to be performed, Mice were anaesthetised and maintained with 2% isofluorane in oxygen then imaged using a 9.4T MRI system (Agilent, Palo Alto, CA, US) and a 38 mm quadrature driven birdcage RF coil (Rapid Biomedical, Rimpar, Germany). Multi-slice cardiac and respiratory gated cine-MRI was performed in the true short-axis orientation and covered the whole left ventricle (LV) [6]. Late gadolinium enhanced (LGE-MRI) was performed 20 minutes after intraperitoneal (i.p.) injection of 0.5 mmol.kg-1 Gd-DTPA-BMA (Omniscan. GE Healthcare, Hatfield, UK), using a multi-slice inversion recovery sequence [8]. Data were analysed in a blinded fashion using ImageJ (NIH, Bethesda, MD, US). Standard measures of left ventricular morphology and function were made from cine stacks, and infarct area determined by thresholding to 3 standard deviations above the mean remote myocardial signal intensity.

The TG2 inhibitor (25 mg/kg/day in 50% DMSO in PBS, pH 7.4, n = 7) or control (50% DMSO in PBS, pH 7.4, n = 5) was delivered to the mice using as an Alzet osmotic minipump (Alzet1002) as described for the Angiotensin II model. The pump was implanted subcutaneously 24 h after induction of the infarct and maintained for 20 days at which time the animals were sacrificed.

Collagen staining in non-infarcted myocardium remote from the scar areas was undertaken as previously reported [9], with collagen content assessed from a minimum of 24 images per mouse including both fibrotic and non-fibrotic areas.

For analysis of the ε(γ-glutamyl)lysine crosslink, tissue samples from both models were digested using a cocktail of proteolytic enzymes and the crosslink then analysed using cation exchange amino acid analysis using a lithium buffer system as previously described [10].

Statistical analysis and power calculations

Unless stated otherwise, all values are presented as the mean ± S.D for at least three independent replicate experiments ($n \geq 3$). Data analyses were performed using either the

Turkey and Dunnet test or the Student's t test. A p value of less than 0.05 was considered to indicate statistical significance when is indicated in the text.

All experiments and analysis of data were conducted in a blind manner with no outliers or data excluded. For animal work and for crosslink content of tissues, statistical analysis of results was undertaken using one-way analysis of variance (ANOVA) using a post-test depending on the requirement. Data are expressed as the mean ± S.E. A p value of less than 0.05 was considered to indicate statistical significance.

For in vivo work, sample size was calculated by conducting a power analysis (using freeware G*Power v3.9.1.2, *http://www.gpower.hhu.de/en.html) taking into account work by Charan and Kantharia* [11] *and Dell et al* [12] *and* utilising standard deviation from previous data[3], and also taking into account the 3Rs (reduction, replacement and refinement). A mandatory requisite for using animals in research as stipulated by national guidelines from national (*Animals Scientific Procedures Act 1986, UK*) and local ethical guidelines. In the AngII model, our previous data showed there was a standard deviation of 0.8. In order to observe a difference of 20% in cardiac fibrosis, the sample size of five was determined using two sided (independent) α of 0.05 with a desired power of 0.80 which will enable to distinguish. A post-hoc power analysis was carried out to determine if further experiments were needed, since one animal randomly died in a group. The death was not attributed to be significant as previous experience has shown that AngII-LVH model has a 1 in 12 mortality rate). Using data for the primary, cardiac fibrosis, inputting a sample number of 4 and 5 with respective S.D. of 0.94 and 0.78 predicted a power of >0.95, confirming there was an adequate power to observe the 50% reduction in cardiac fibrosis, and therefore no scientific justification to increase sample number.

Based on our extensive previous data with the mouse MI model [7], we have found that the most commonly used clinical end points of ejection fraction and infarct size have a standard

deviation of 5% when measured using MRI, meaning that to detect a therapy mediated improvement in ejection fraction from 25 to 35%, or reduction in infarct size from 35 to 25% (power 80%, $p < 0.05$), a minimum of 4 animals would be required. This also takes into account the 3R's (reduction, replacement and refinement) as a mandatory requisite for using animals in research as stipulated by national guidelines from national (*Animals (Scientific Procedures) Act 1986, UK*) and local ethical guidelines. Power calculations were performed as defined by Charan and Kantharia [11] and Dell et al [12].

***In vitro* studies**

Cells

HUVECs (Lonza, Germany) were cultured in complete EGM endothelial cell culture medium (Lonza, Belgium). Human cardiofibroblasts and culture medium were purchased from PromoCell (Germany).

TG2-/- and control TG2+/+ ECs were isolated from 4-6 weeks old B6 TG2 ko or wt mice [13]. The lung tissue was removed and incubated with 5ml of collagenase (0.5mg/ml) with gentle agitation for 30 min at 37ºC. After filtering through the 100µm nylon-cell strainer, the cell were pelleted at 800 rpm for 10 min at 4ºC and washed once with RPMI containing 5% FBS. The cell pellet was re-suspended with 30% Opti-Prep solution (Progen Biotechnik, Germany) with the uplayer of 2 ml 5% FBS RPMI and centrifugated at 3000 rpm for 20 min at 4ºC. The cell fraction in RPMI solution was gently washed with 5ml of the washing buffer and centrifuged at 800 rpm for 10 min at 4ºC. The cell pellet was purified using anti-CD102 antibody and subsequently anti-CD31 antibody using magnetic beads as introduced before [14]. The isolated endothelial cells were cultured at 37 °C in a humidified environment at 5% CO_2.on the collagen I pre-coated tissue culture plates in complete EGM endothelial medium and then used for future experiments.

Cell viability assay

The XTT assay was used to measure viable cell numbers as described previously [15].

Angiogenesis assay using a V2a Angiokit angiogenesis co-culture system

The V2a AngioKit assay a prefrozen form of the AngioKit assay (TCS Cellworks) was used to study microtubule formation in co-cultures of HUVECs and primary human fibroblasts [16]. Following seeding, medium was replaced at Day 2 by fresh Growth Medium and changed every two days over 9-14 days. At Day 2, treatments were introduced to the cells as further described in the figures and figure legends. To visualize the microtubule structures, cells were fixed in cold Ethanol (70%) at room temperature for 30 min and stained with the anti-human CD31 primary antibody for 1 h at 37°C and detected using the goat anti-mouse IgG alkaline phosphatise (ALP)-conjugate secondary antibody. Microtubules were revealed using ALP soluble substrate p-nitrophenol phosphate. Microtubules were finally stained using BCIP/NBT substrate for 15-20 min at 37°C. The images were photographed with a 10× objective using bright-field microscopy (Nikon, UK) and microtubule formation quantified using the TCS Cellworks AngioSys Image Analysis Software (ZHA-1800).

Biotinylation of cell surface protein

Cell surface proteins of human cardiofibroblasts were biotin-labelled as described previously [17]. Following treatments, human cardiofibroblast monolayers were incubated with 0.8 mM sulfo-NHS-LC-biotin dissolved in PBS, pH 8.0, at 4°C for 20 min and lysed with 1% SDS at 4°C in PBS, pH 8.0. Following denaturing at 95°C, the cell lysates were pre-cleared by centrifugation at 14,000 ×g and 200 µg of total protein was incubated with NeutrAvidin-agarose resin overnight at 4°C. Following three washes with PBS, pH 8.0, the biotin-labelled proteins were dissolved in 2× Laemmli buffer analysed for TG2 using Western blotting.

Co-immunoprecipitation (Co-IP)

Co-IP was performed to detect the direct interaction between TG2 and syndecan-4 [17]. Briefly, Human cardiofibroblasts, following treatments, were lysed in co-IP buffer and 0.5 µg of anti-

syndecan-4 antibody was used to pull down the syndecan-4 immunocomplex. Western blotting was then carried out to detect the presence of TG2 in this complex.

Western Blot and immunofluorescence staining

Following treatments, cells were lysed in cell lysis buffer and used for SDS-PAGE and Western blotting as described previously [18]. For the ECM fractions, the cells were lifted using 2mM EDTA in PBS, pH 7.4. Remaining matrices were collected into Laemmli buffer [17]. For the cellular fractions, the membranes were re-probed with GAPDH as the loading control [19]. Signals were detected using the SynGene system. Densitometry was performed using the ImageJ software and band intensity normalised to GAPDH taken from 3 separate experiments. Where differences are statistically different (p <0.05) this is indicated in the text. The mean densitometry values ± S.D. are shown in the Supplementary figures.

Immunofluorescence staining was performed as detailed by Jones *et al* [26] to detect the presence of extracellular collagen deposited by human cardiofibroblasts and VE-cadherin in HUVECs. Briefly, following treatments, the cells were fixed with 3.7% paraformaldehyde in PBS, pH 7.4 for 15 min at room temperature. Following blocking with 3% BSA in PBS, pH 7.4 (blocking buffer), for 30 min at 37°C, primary antibodies (1:100 dilution in blocking buffer) were incubated with the cells for 2 h at 37°C, followed by 2 h incubation with fluorescence-labelled secondary antibodies at 37°C. The cells were mounted in the mount medium containing DAPI and visualized using an epi-fluorescence microscope. Image processing included changes in brightness, contrast and tonal range, and was applied equally across the entire image. ImageJ software was used to measure the pixels of the fluorescence signal for quantification purposes.

References

1. Badarau E, Mongeot A, Collighan R, Rathbone D, Griffin M. Imidazolium-based warheads strongly influence activity of water-soluble peptidic transglutaminase inhibitors. *European journal of medicinal chemistry*. 2013;66:526-530
2. Murdoch CE, Alom-Ruiz SP, Wang M, Zhang M, Walker S, Yu B, Brewer A, Shah AM. Role of endothelial nox2 nadph oxidase in angiotensin ii-induced hypertension and vasomotor dysfunction. *Basic research in cardiology*. 2011;106:527-538
3. Murdoch CE, Chaubey S, Zeng L, Yu B, Ivetic A, Walker SJ, Vanhoutte D, Heymans S, Grieve DJ, Cave AC, Brewer AC, Zhang M, Shah AM. Endothelial nadph oxidase-2 promotes interstitial cardiac fibrosis and diastolic dysfunction through proinflammatory effects and endothelial-mesenchymal transition. *Journal of the American College of Cardiology*. 2014;63:2734-2741
4. Badarau E, Wang Z, Rathbone DL, Costanzi A, Thibault T, Murdoch CE, El Alaoui S, Bartkeviciute M, Griffin M. Development of potent and selective tissue transglutaminase inhibitors: Their effect on tg2 function and application in pathological conditions. *Chemistry & biology*. 2015;22:1347-1361
5. Jang HS, Lee HC, Chin KB. Effects of red bean (vigna angularis) protein isolates on rheological properties of microbial transglutaminase mediated pork myofibrillar protein gels as affected by fractioning and preheat treatment. *Korean journal for food science of animal resources*. 2016;36:671-678
6. Stuckey DJ, Carr CA, Tyler DJ, Aasum E, Clarke K. Novel mri method to detect altered left ventricular ejection and filling patterns in rodent models of disease. *Magnetic resonance in medicine*. 2008;60:582-587
7. Noseda M, Harada M, McSweeney S, Leja T, Belian E, Stuckey DJ, Abreu Paiva MS, Habib J, Macaulay I, de Smith AJ, al-Beidh F, Sampson R, Lumbers RT, Rao P, Harding SE, Blakemore AI, Jacobsen SE, Barahona M, Schneider MD. Pdgfralpha

demarcates the cardiogenic clonogenic sca1+ stem/progenitor cell in adult murine myocardium. *Nature communications*. 2015;6:6930

8. Stuckey DJ, McSweeney SJ, Thin MZ, Habib J, Price AN, Fiedler LR, Gsell W, Prasad SK, Schneider MD. T(1) mapping detects pharmacological retardation of diffuse cardiac fibrosis in mouse pressure-overload hypertrophy. *Circulation. Cardiovascular imaging*. 2014;7:240-249
9. Nisbet AM, Camelliti P, Walker NL, Burton FL, Cobbe SM, Kohl P, Smith GL. Prolongation of atrio-ventricular node conduction in a rabbit model of ischaemic cardiomyopathy: Role of fibrosis and connexin remodelling. *Journal of molecular and cellular cardiology*. 2016;94:54-64
10. Griffin M, Wilson J, Lorand L. High-pressure liquid chromatographic procedure for the determination of epsilon-(gamma-glutamyl)lysine in proteins. *Analytical biochemistry*. 1982;124:406-413
11. Charan J, Kantharia ND. How to calculate sample size in animal studies? *Journal of pharmacology & pharmacotherapeutics*. 2013;4:303-306
12. Dell RB, Holleran S, Ramakrishnan R. Sample size determination. *ILAR journal*. 2002;43:207-213
13. Tong L, Png E, Aihua H, Yong SS, Yeo HL, Riau A, Mendoz E, Chaurasia SS, Lim CT, Yiu TW, Iismaa SE. Molecular mechanism of transglutaminase-2 in corneal epithelial migration and adhesion. *Biochimica et biophysica acta*. 2013;1833:1304-1315
14. Wang ZP, M.; Lee, E-S.; Kojima, S.; Griffin, M. The functional relationship between transglutaminase 2 and transforming growth factor β1 in the regulation of angiogenesis and endothelial-mesenchymal transition. *Cell death & disease*. 2017

15. Kotsakis P, Wang Z, Collighan RJ, Griffin M. The role of tissue transglutaminase (tg2) in regulating the tumour progression of the mouse colon carcinoma ct26. *Amino acids*. 2011;41:909-921

16. Bishop ET, Bell GT, Bloor S, Broom IJ, Hendry NF, Wheatley DN. An in vitro model of angiogenesis: Basic features. *Angiogenesis*. 1999;3:335-344

17. Wang Z, Collighan RJ, Pytel K, Rathbone DL, Li X, Griffin M. Characterization of heparin-binding site of tissue transglutaminase: Its importance in cell surface targeting, matrix deposition, and cell signaling. *The Journal of biological chemistry*. 2012;287:13063-13083

18. Nadella V, Wang Z, Johnson TS, Griffin M, Devitt A. Transglutaminase 2 interacts with syndecan-4 and cd44 at the surface of human macrophages to promote removal of apoptotic cells. *Biochimica et biophysica acta*. 2015;1853:201-212

19. Wang Z, Perez M, Caja S, Melino G, Johnson TS, Lindfors K, Griffin M. A novel extracellular role for tissue transglutaminase in matrix-bound vegf-mediated angiogenesis. *Cell death & disease*. 2013;4:e808
