# Supplementary material for: Cardiac fibrosis can be attenuated by blocking the activity of transglutaminase 2 using a selective small-molecule inhibitor
Source: Cell Death Dis. 2018 Apr 27;9(6):613. doi: 10.1038/s41419-018-0573-2 (PMC5966415; doi:10.1038/s41419-018-0573-2)
Supplement: Supplementary file 3 — Supplementary Files-Supplementary Figure 2 [file 41419_2018_573_MOESM3_ESM.pdf]

Supplementary Files-Supplementary Figure S2

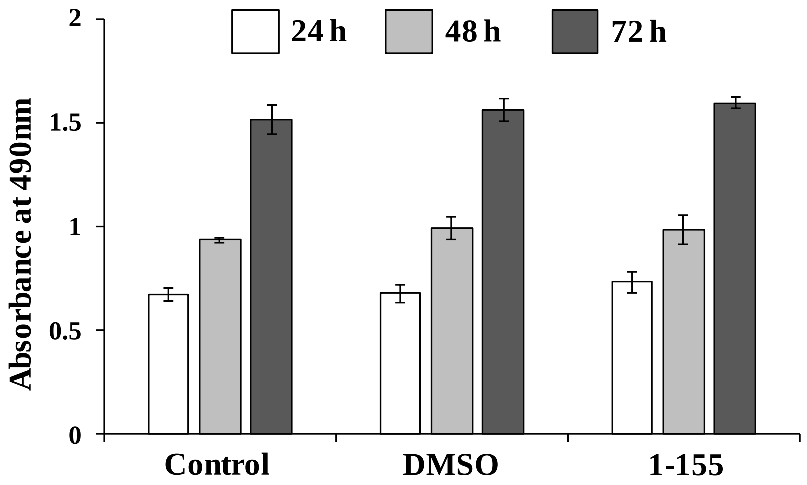

Supplementary Figure S2. Effect of TG2 inhibitor 1-155 on the growth of Cardiofibroblasts. Cardiofibroblasts were seeded into 96-well plates at the density of 3,000 cells/well. TG2-specific inhibitor 1-155 (at 2.5μM) was used to treat the cells, while DMSO was used as the vehicle control treatment. The XTT assay was performed at 24, 48 and 96 hours incubation period to measure cell viability. Data are the means ± S.D. from 3 separate experiments.
