# Supplementary material for: Cardiac fibrosis can be attenuated by blocking the activity of transglutaminase 2 using a selective small-molecule inhibitor
Source: Cell Death Dis. 2018 Apr 27;9(6):613. doi: 10.1038/s41419-018-0573-2 (PMC5966415; doi:10.1038/s41419-018-0573-2)
Supplement: Supplementary file 5 — Supplementary Files-Supplementary Figure 4 [file 41419_2018_573_MOESM5_ESM.pdf]

Supplementary Files-Supplementary Figure S4

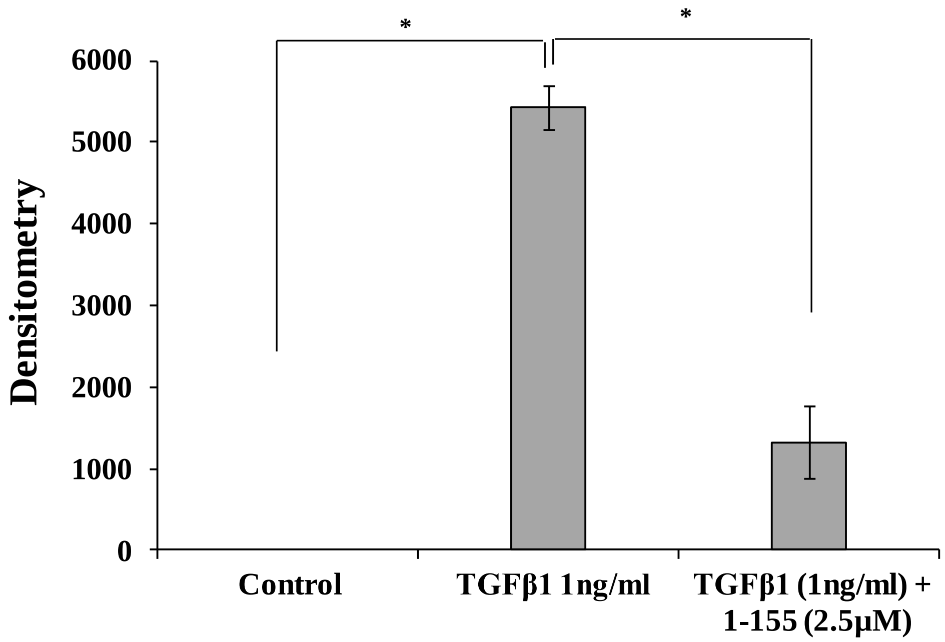

Supplementary Figure S4. Densitometry of the Western blots for TG2 present in the syndecan-4 immuno-complex as shown in **Figure 4c**. Data are the means ± S.D. from 3 separate experiments. *, $p<0.05$.
