# Supplementary material for: Cardiac fibrosis can be attenuated by blocking the activity of transglutaminase 2 using a selective small-molecule inhibitor
Source: Cell Death Dis. 2018 Apr 27;9(6):613. doi: 10.1038/s41419-018-0573-2 (PMC5966415; doi:10.1038/s41419-018-0573-2)
Supplement: Supplementary file 6 — Supplementary Files-Supplementary Figure 5 [file 41419_2018_573_MOESM6_ESM.pdf]

Supplementary Files-Supplementary Figure S5

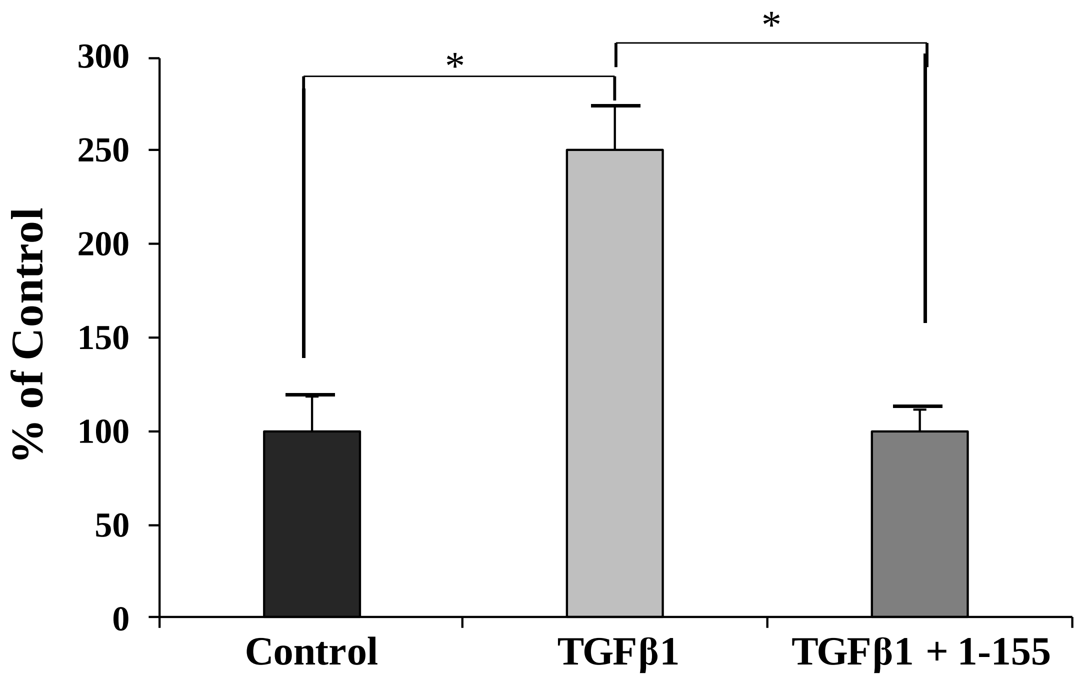

Supplementary Figure S5. Quantification of the inhibition of collagen deposition in TGFβ1 treated cardiofibroblasts by TG2 inhibitor 1-155. Changes in collagen deposition were quantified via measuring the fluorescence signal. Changes in brightness, contrast and tonal range were applied equally across the entire image. ImageJ software was used to measure the pixels of the fluorescence signal for quantification purposes. Data was collected from at least 5 separate areas from 2 separate experiments. The control group was used as 100% and used to normalized data collected from the experimental groups. *, $p<0.05$.
