# Supplementary material for: Cardiac fibrosis can be attenuated by blocking the activity of transglutaminase 2 using a selective small-molecule inhibitor
Source: Cell Death Dis. 2018 Apr 27;9(6):613. doi: 10.1038/s41419-018-0573-2 (PMC5966415; doi:10.1038/s41419-018-0573-2)
Supplement: Supplementary file 8 — Supplementary Files-Supplementary Figure 7 [file 41419_2018_573_MOESM8_ESM.pdf]

Supplementary Files-Supplementary Figure S7

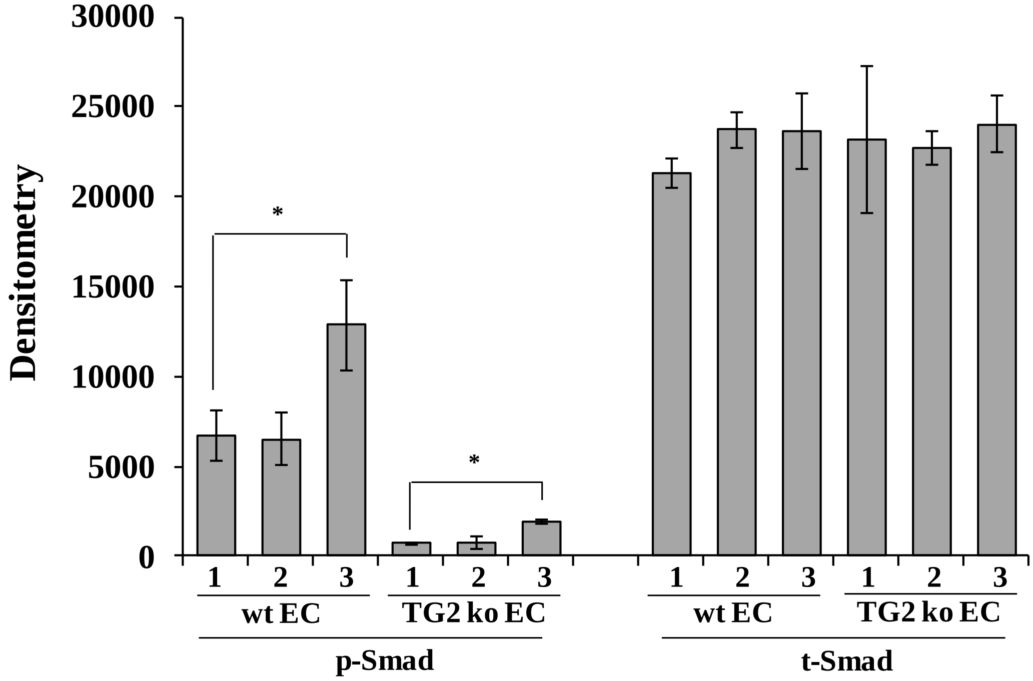

Supplementary Figure S7. Densitometry of the Western blots of phosphorylated Smad2/3 (p-Smad2/3) and total Smad (t-Smad2/3) in wild type and TG2 KO EC as shown in **Figure 5c**. Lane 1: Control; Lane 2: TGFβ1 treatment at 1ng/ml; and Lane 3: TGFβ1 treatment at 10ng/ml. Data are the means ± S.D. from 3 separate experiments. *, p<0.05.
