# Supplementary material for: Cardiac fibrosis can be attenuated by blocking the activity of transglutaminase 2 using a selective small-molecule inhibitor
Source: Cell Death Dis. 2018 Apr 27;9(6):613. doi: 10.1038/s41419-018-0573-2 (PMC5966415; doi:10.1038/s41419-018-0573-2)
Supplement: Supplementary file 9 — Supplementary Files-Supplementary Figure 8 [file 41419_2018_573_MOESM9_ESM.pdf]

Supplementary Files-Supplementary Figure S8

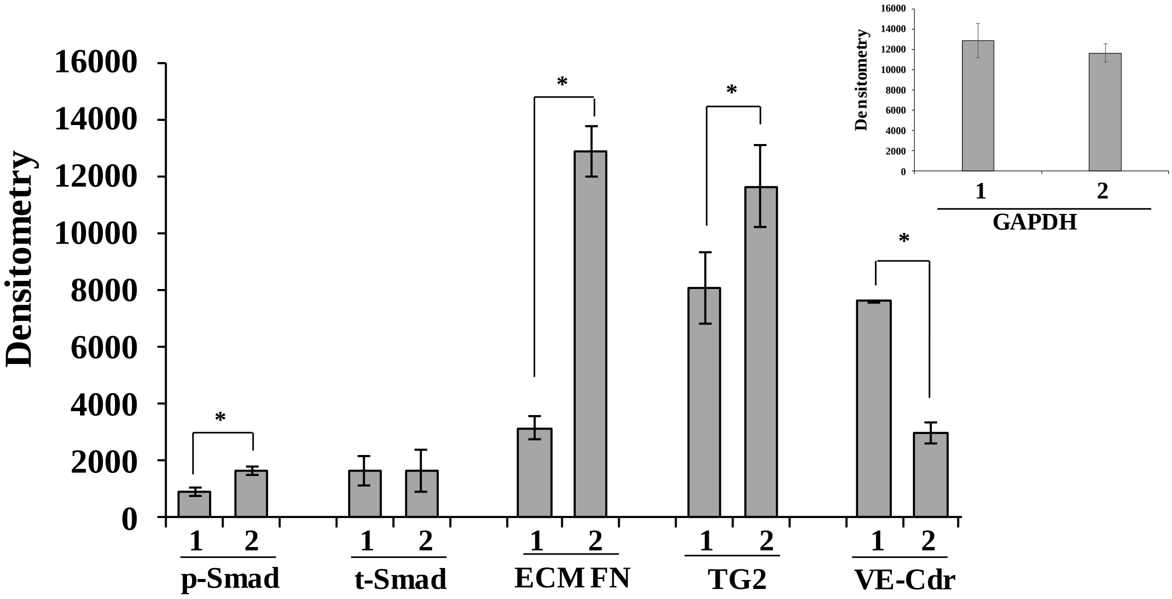

Supplementary Figure 8. Densitometry of the Western blots of phosphorylated Smad2/3 (p-Smad2/3), total Smad (t-Smad2/3), ECM FN, TG2 and VE-cadherin (VE-Cdr) in HUVEC as shown in **Figure 5d**. GAPDH was used as the equal loading control. Lane 1: Control; and Lane 2: Thioredoxin at 2.5µM. Data are the means ± S.D. from 3 separate experiments. *, $p<0.05$.
