# Supplementary material for: Cardiac fibrosis can be attenuated by blocking the activity of transglutaminase 2 using a selective small-molecule inhibitor
Source: Cell Death Dis. 2018 Apr 27;9(6):613. doi: 10.1038/s41419-018-0573-2 (PMC5966415; doi:10.1038/s41419-018-0573-2)
Supplement: Supplementary file 10 — Supplementary Files-Supplementary Table 1 [file 41419_2018_573_MOESM10_ESM.pdf]

Supplementary Files-Table 1

Supplementary Table 1. List of antibodies and suppliers used in the study

**Antigen**	**Species source**	**Company**	**Comments**
TG2 (Clone Cub7402+TG100	Mouse, monoclonal	Lab Vision (Thermo Fisher, UK)	
VE-Cadherin	Rabbit, polyclonal	Santa Cruz, USA	Endothelial marker
p-Smad2/3	Rabbit, polyclonal	Cell Signalling Technology,	TGFβ signalling
t-Smad2/3	Rabbit, polyclonal	Cell Signalling Technology,	
αSMA	Rabbit, polyclonal	Cell Signalling Technology,	
FN	Rabbit, polyclonal	Sigma-Aldrich, UK	
Syndecan-4	Rabbit, polyclonal	Fisher, UK	
Collagen I	Rabbit, polyclonal	Sigma-Aldrich, UK	
GAPDH	Rabbit, polyclonal	Santa Cruz, USA	Equal loading
Anti-mouse secondary and		Dako (Denmark)	
