# Supplementary material for: Cardiac fibrosis can be attenuated by blocking the activity of transglutaminase 2 using a selective small-molecule inhibitor
Source: Cell Death Dis. 2018 Apr 27;9(6):613. doi: 10.1038/s41419-018-0573-2 (PMC5966415; doi:10.1038/s41419-018-0573-2)
Supplement: Supplementary file 11 — Supplementary Files-Supplementary Table 2 [file 41419_2018_573_MOESM11_ESM.pdf]

Supplementary Files-Table 2

[1]Supplementary Table 2. Effect of TGFβ on tubule formation of HUVECs in the co-culture *in vitro* model of angiogenesis

Treatment	Length	Junctions	Branches	Mean Branch Length
**Control**	7145.7 ± 641.5	258.2 ± 33.6	337.7 ± 35.3	21.5 ± 2.1
**TGFβ1**	2266.0 ± 24.3[a]	58.3 ± 14.4[a]	91.0 ± 24.3[a]	29.7 ± 1.5

[1]Calibration= 1 pixel

[a] Significantly different vs Control ($p<0.05$)

Supplementary Table 1. The effect of exogenous TGFβ on tubule formation in angiogenesis co-cultures. TGFβ1 (1ng/ml) was applied to the Angio V2a co-cultures during 12-day culture period. Microtubule formation as shown in **Fig. 2B** was quantified using the TCS Cellworks AngioSys Image Analysis Software. Data represent Mean values ± S.D. from 3 separate experiments
